# Chronic kidney disease with genitourinary tuberculosis: old disease but ongoing complication

**DOI:** 10.1186/s12882-018-0994-2

**Published:** 2018-08-02

**Authors:** Eun Jin Kim, Woonji Lee, Woo Yong Jeong, Hen Choi, In Young Jung, Jin Young Ahn, Su Jin Jeong, Nam Su Ku, Jun Yong Choi, Young Hwa Choi, Young Goo Song, June Myung Kim

**Affiliations:** 1 Department of Infectious Diseases, Ajou University School of Medicine, 164, World cup-ro, Yeongtong-gu, Suwon-si, Gyeonggi-do 16499 Republic of Korea; 2 Division of Infectious Disease, Department of Internal Medicine, Yonsei University College of Medicine, 50-1 Yonsei-ro, Seodaemun-gu, Seoul, 120-752 Republic of Korea; 3 AIDS Research Institute, Yonsei University College of Medicine, Seoul, Republic of Korea

**Keywords:** Genitourinary tuberculosis, Chronic kidney disease, Risk factor

## Abstract

**Background:**

Genitourinary tuberculosis (GUTB) is a type of extrapulmonary TB that exerts a deleterious effect on renal function by promoting renal calcification and ureteric stricture. Therefore, we investigated the risk factors for chronic kidney disease (CKD) in GUTB patients after the end of treatment.

**Methods:**

This retrospective study was conducted at a tertiary hospital in South Korea. Data from patients (>18 years of age) with GUTB were collected from January 2005 to July 2016. CKD was defined as a glomerular filtration rate <60 mL/min/1.73m^2^ after the end of treatment.

**Results:**

In total, 56 patients with GUTB (46.4% males; mean age 52.8 ± 16.6 years) were enrolled in the study. CKD developed in 11 (19.6%) patients and end-stage renal disease in 4 (7.1%). In a univariate analysis, older age (*p* = 0.029), microscopic haematuria (*p* = 0.019), proteinuria (*p* = 0.029), acute renal failure (ARF) (*p* < 0.001) and a positive polymerase chain reaction-based test result for TB in the urine (*p* = 0.030) were significantly associated with decreased renal function. In a multivariate analysis, ARF (odds ratio [OR], 54.31; 95% confidence interval [CI], 1.52–1944.00; *p* = 0.032) and old age (OR, 54.26; 95% CI, 1.52–1932.94; *p* = 0.028) were independent risk factors for CKD in GUTB patients.

**Conclusions:**

ARF and old age were independent risk factors for CKD in GUTB patients. Therefore, in elderly GUTB patients with ARF at the time of diagnosis, regular follow-up of renal function should be performed even after the end of treatment.

## Background

Tuberculosis (TB) is an important unresolved public health issue, affecting millions of people each year. In 2015, TB was one of the top 10 infectious causes of death worldwide; there were 10.4 million new TB cases, equivalent to 142 cases per 100,000 population, and 1.4 million deaths due to TB, with an additional 0.4 million deaths resulting from TB among human immunodeficiency virus (HIV)-positive persons [1]. South Korea has an intermediate burden of TB; according to the Korea Center for Disease Control, the estimated TB incidence is 63.2 cases per 100,000 population (in 2015, *n* = 32,181). Extrapulmonary TB (EPTB) accounted for 13.0% of all new cases reported in 2015 (*n* = 6631). Genitourinary TB (GUTB) is a type of EPTB that infected 199 individuals in South Korea in 2015, accounting for 3.0% of all EPTB cases [2, 3].

Although GUTB is a rare form of EPTB, it is an important cause of progressive chronic kidney disease (CKD). The kidneys are the most common site of GUTB, with bacteria spreading haematogenously. Tuberculous bacilli can lead to granuloma formation in glomeruli and entry into the medullary interstitium. Subsequently, renal papilla destruction can develop due to calyceal ulceration and involvement of the collecting system. This destruction may extend towards the urothelium and induce stricture formation, resulting in hydroureter and hydronephrosis. Renal calcification in GUTB is common, and patients with renal TB can develop bladder contracture. In addition, TB can affect the male and female genital tracts [4, 5]. GUTB induces end-stage renal disease (ESRD) in 5.7% of patients [5]. According to the European Dialysis and Transplant Association registry, 0.65% of new dialysis cases are caused by renal TB [6]. In Korea, there were 14,756 new dialysis patients in 2015, of whom 0.1% required dialysis because of GUTB [7, 8].

However, in a previous autopsy study, only 50% of patients with GUTB were symptomatic, and only 18% had received a clinical diagnosis [5]. Additionally, the clinical manifestations of GUTB are nonspecific [9]. Thus, the diagnosis is often delayed, during which GUTB progression may lead to CKD due to parenchymal destruction and obstructive uropathy. Therefore, we investigated the characteristics of urogenital TB in adult patients with no history of CKD in a single-centre retrospective observational study and identified risk factors for CKD development after ending GUTB treatment.

## Methods

### Study population and design

We conducted this retrospective study at the Severance Hospital, a 2400-bed university-affiliated teaching hospital and tertiary care referral hospital in Seoul, South Korea. We enrolled participants older than 18 years of age diagnosed with GUTB. We identified patients with ICD-10 codes A18.1, B90.1 and N33.0 from January 2005 to July 2016 from electronic medical records and enrolled only those who started and completed the treatment during the study period. Clinical and laboratory data at the time of GUTB diagnosis, including age, sex, medical history, follow-up duration and symptoms, were collected. To assess renal function, serum creatinine levels were reported before treatment and at the 6- and 12-month post-treatment follow-ups. Body mass index (BMI) was calculated as weight divided by height squared (kg/m^2^). We also investigated the diagnostic methods, treatment modalities and outcomes of GUTB patients during a recent 10-year period in one centre in Korea to identify risk factors for CKD developing after ending treatment. All surgical techniques were investigated included all ablative surgery and reconstructive surgery between diagnosis and during medical treatment. We excluded patients with a follow-up duration of <1 year, unfulfilled GUTB diagnostic criteria, pre-existing CKD and insufficient data. This study was approved by the Institutional Review Board of the Yonsei University Health System Clinical Trial Center.

### Definitions

The diagnosis of GUTB was defined as the presence of any clinical finding plus a positive result for one of the following: (1) acid-fast bacilli (AFB) in urine, (2) urine culture of *Mycobacterium tuberculosis* (*M. tuberculosis*), (3) polymerase chain reaction (PCR) for *M. tuberculosis* in urine or (4) histopathological evidence of TB in any GU tissue specimen. A histological diagnosis of TB was confirmed by identifying caseating necrosis, loose aggregates of epithelioid histiocytes and Langerhans giant cells in tissue specimens [6, 10]. Immunosuppressant use was defined as a daily dose of ≥20 mg prednisolone-equivalent steroid, monoclonal antibodies, antimetabolite drugs or T-cell inhibitors within 30 days prior to diagnosis of GUTB. Pre-TB (pre-existing) CKD was defined as an estimated glomerular filtration rate (eGFR) of <60 mL/min/1.73m^2^ for more than 3 months before diagnosis of GUTB or a self-reported history. Acute renal failure (ARF) was defined according to the KDIGO (Kidney Disease: Improving Global Outcomes) criteria (increased serum creatinine level ≥ 1.5-fold compared with baseline or by ≥0.3 mg/dL). We used the serum creatinine level to investigate the presence of ARF at the time of GUTB diagnosis. The eGFR was calculated from the serum creatinine level using the Modification of Diet in Renal Diseases (MDRD) equation [11]. Microscopic haematuria was defined as the excretion of more than two red blood cells per high-power field in a centrifuged urine specimen and pyuria as the excretion of more than five white blood cells. Proteinuria was defined as more than one positive urine dipstick test. Anaemia was defined as a haemoglobin level <11 g/dL according to the KDIGO guidelines based on the target for renal anaemia therapy [12].

### Decreased kidney function and poor outcome

CKD group was defined as an eGFR <60 mL/min/1.73m^2^ after completing GUTB treatment. We used the serum creatinine level between 6 months and 1 year after completion of GUTB treatment to investigate the presence of CKD. Patients with a history of CKD at the time of GUTB diagnosis (pre-TB CKD) were excluded. To assess kidney function, serum creatinine levels were determined before treatment and at the 6- and 12-month post-treatment follow-ups. After the treatment was completed, any surgical treatment, recurrence, ESRD development, and all cause mortality were investigated.

### Statistical analysis

All statistical analyses were performed using the Statistical Package for the Social Sciences for Windows (ver. 23.0, SPSS Inc., Chicago, IL, USA). To identify risk factors for CKD, parameters were compared between patients who did and those who did not develop CKD. Continuous variables are expressed as means ± standard deviation and categorical variables as numbers (percentages). The Kolmogorov–Smirnov test was used to analyse the normality of the data distribution. Non-normally distributed data are expressed as medians and interquartile ranges (IQRs). We used Student’s *t*-test or Mann–Whitney U-test and the χ^2^ test or Fisher’s exact test to compare continuous and categorical variables, respectively, in univariate analyses. Variables with *p*-values <0.05 by the Wald test in the univariate analyses were included in a multivariate logistic regression analysis to identify risk factors for CKD. The multivariate analysis results are expressed as odds ratios (ORs) and 95% confidence intervals (CIs). A two-sided *p* value <0.05 was considered to indicate significance.

## Results

### Demographic characteristics

**Fig. 1:**
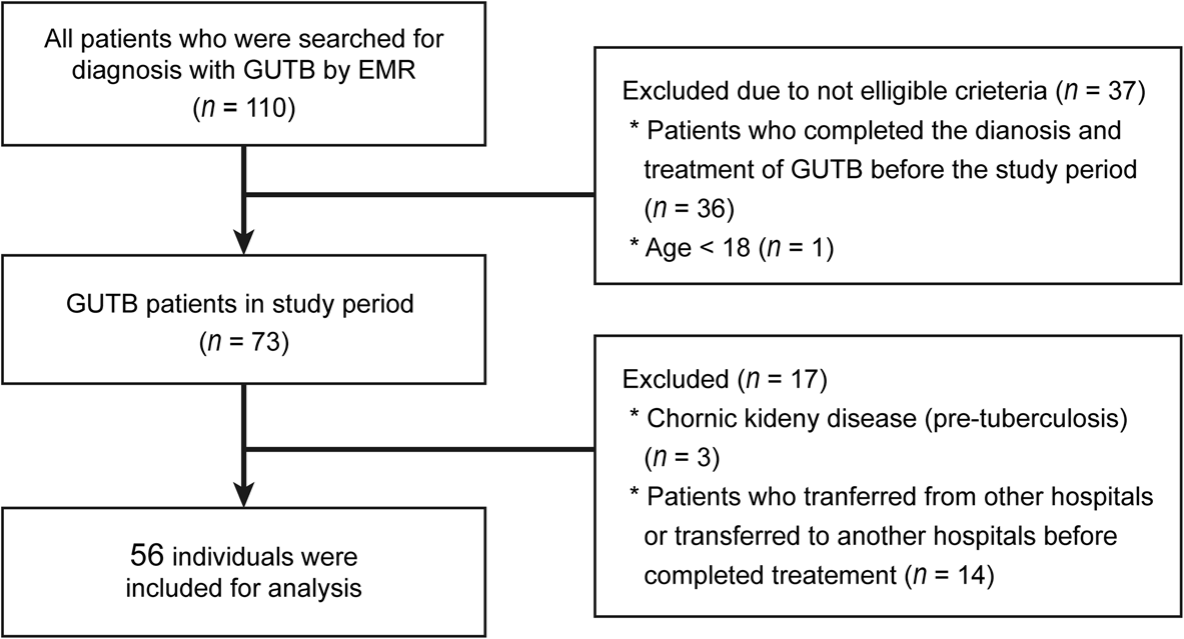
Inclusion and exclusion criteria applied in this study. *GUTB* genitourinary tuberculosis, *EMR* electronic medical record

During a 10-year period, 56 patients were diagnosed with GUTB, of whom 11 (19.6%) developed CKD after treatment for GUTB (Fig. 1). The mean age of these patients was 52.8 years, and 24 (42.9%) patients had a history of TB, most frequently pulmonary TB (21/24). Three patients were taking immunosuppressants, and two had a history of gastrectomy. No patient had HIV or multi-drug resistant TB. The most frequent symptom was abdominal pain (42.9%), and gross haematuria was present in 33% of the patients (Table 1).

**Table 1:** Baseline characteristics of both groups according to kidney function in patients with GUTB

Variables	Normal kidney function group (*n* = 45)	CKD group (*n* = 11)	Total (*n* = 56)	*p*
Age	50.1 ± 15.1	63.9 ± 18.5	52.8 ± 16.6	**0.012**
Old age (≥65 years)	6 (13.3)	5 (45.5)	11 (19.6)	**0.029**
Sex (male)	20 (44.4)	6 (54.5)	26 (46.4)	0.609
BMI (kg/m^2^)	22.8 ± 3.4	21.9 ± 5.2	22.6 ± 3.8	0.589
Previous TB history				
Lung	16 (35.6)	5 (45.5)	21 (37.5)	0.730
Bone	2 (4.4)	1 (9.1)	3 (5.4)	0.488
Intestine	1 (2.2)	0 (0.0)	1 (1.8)	1.000
Co-morbidity conditions				
Immune-compromised patients	8 (17.8)	4 (36.4)	12 (21.4)	0.224
Cardiovascular disease	11 (24.4)	5 (45.5)	16 (28.6)	0.312
DM	1 (2.2)	2 (18.2)	3 (5.4)	0.174
Liver disease	3 (6.7)	1 (9.1)	4 (7.1)	1.000
Pulmonary disease	4 (8.9)	2 (18.2)	6 (10.7)	0.727
History of gastrectomy	2 (4.4)	0 (0.0)	2 (3.6)	1.000
Involving location				
Urinary system				
Kidney or ureter	30 (66.7)	9 (81.8)	39 (69.6)	0.539
Bladder	11 (24.4)	5 (45.5)	16 (28.6)	0.312
Genital system				
Epididymis or testis	11 (24.4)	2 (18.2)	13 (23.2)	0.966
Prostate	4 (8.9)	0 (0.0)	4 (7.1)	0.709
Uterus or fallopian tubes	4 (8.9)	1 (9.1)	5 (8.9)	1.000
Both affected urinary and genital tract	2 (4.4)	0 (0.0)	2 (3.6)	1.000
Concurrent extra-urogenital lesion	1 (2.2)	1 (9.1)	2 (3.6)	0.132
Clinical features				
Nonspecific symptoms^a^	5 (11.1)	2 (18.2)	7 (12.5)	0.614
Urinary symptoms^b^	22 (48.9)	9 (81.8)	31 (55.4)	0.088
Gross hematuria	14 (31.1)	5 (45.5)	19 (33.9)	0.481
Loin/abdominal pain	18 (40.0)	6 (54.5)	24 (42.9)	0.501
Scrotal pain/mass	11 (24.4)	0 (0.0)	11 (19.6)	0.098
Abscess or fistula	2 (4.4)	1 (9.1)	3 (5.4)	0.488
Vaginal bleeding	2 (4.5)	0 (0.0)	2 (3.6)	1.000
Asymptomatic patients	4 (8.9)	0 (0.0)	4 (7.1)	0.575

Bold values indicate statistically significant differences

Data were presented as mean ± SD, number (percentage) or median (IQR)

*GUTB* genitourinary tuberculosis, *CKD* chornic kidney disease, *BMI* body mass index, *TB* tuberculosis, *DM* diabetes mellitus, *SD* standard deviation, *IQR* interquartile range

^a^Fever; anorexia/weight loss; sweating; weakness; peripheral lymphadenopathy

^b^Urinary frequency or dysuria; urethral pain; irritable voiding symptoms

The diagnosis was confirmed most frequently by histopathology (30/56, 53.6%). Urine AFB staining was positive in six of the 56 patients (10.7%). A TB interferon-gamma evaluation was performed in 5/56 patients, all of whom showed a positive result.

Almost all of the patients (53/56, 94.6%) underwent a 6- or 9-month course of chemotherapy with isoniazid, rifampicin, and ethambutol with or without pyrazinamide; the median treatment duration was 9 months. In addition, 42 of the 56 patients (75.0%) underwent surgical treatment. All surgical techniques included all ablative surgery and reconstructive surgery from the time of diagnosis to completion of medical treatment. During an average 40-month observation period, CKD developed in 11 (19.6%) and ESRD in 4 (7.1%) of the patients. Of the patients, 26.8% required additional surgical treatment after completion of medical TB treatment, and recurrence was observed in 4 (7.1%) patients. The all-cause mortality rate was 7.1% (4/56).

### Risk factors for CKD

CKD occurred in 11 (19.6%) patients during the follow-up period. In univariate analyses, patients with CKD were older (50.1 ± 15.1 vs. 63.9 ± 18.5 years; *p* = 0.012) and had higher creatinine levels at the time of diagnosis (0.9 [IQR 0.8–1.0] vs. 1.4 [IQR 1.1–1.8] mg/dL; *p* = 0.002). ARF occurred in 10 of the 56 patients (17.9%), of whom three recovered and seven progressed to CKD due to persistent renal insufficiency.

There were significantly lower rates of microscopic haematuria (42.2% vs. 81.8%; *p* = 0.019) and proteinuria (13.3% vs. 45.5%; *p* = 0.029) in the non-CKD group compared with the CKD group in the univariate analyses. There was also a significantly higher rate of positive urine TB PCR results in the CKD group (72.7% vs. 37.8%; *p* = 0.030) (Table 2). In the CKD group, five patients had both microscopic haematuria and proteinuria, four had only microscopic haematuria, and two patients had neither microscopic haematuria nor proteinuria. Microscopic haematuria and proteinuria were significant (microscopic haematuria, *p* = 0.019; proteinuria, *p* = 0.029) in the univariate analyses but not in the multivariate analysis.

**Table 2:** Findings of both groups according to kidney function in patients with GUTB

Variables	Normal kidney function group (*n* = 45)	CKD group (*n* = 11)	Total (*n* = 56)	*p*
Diagnosis				
Positivity of urine AFB stain	3/41 (7.3)	3/10 (30.0)	6/51 (11.8)	0.081
Positivity of urinary TB culture	17/38 (44.7)	7/10 (70.0)	24/48 (50.0)	0.155
Positivity of urine TB PCR study	17/36 (47.2)	8/9 (88.9)	25/45 (55.6)	**0.030**
Histopathologic diagnosis	24 (53.3)	6 (54.5)	30/55 (53.6)	0.741
Laboratory data				
White blood cell (/μl)	6880 (5650–7890)	6520 (5580–8395)	6695 (5580–7915)	0.699
Hemoglobin (g/dl)	12.5 ± 1.8	11.7 ± 1.7	12.3 ± 1.8	0.174
Platelet (10^3^/μl)	259.0 ± 80.5	263.2 ± 121.2	259.9 ± 89.1	0.893
ESR (mm/hr)	46.9 ± 23.8	82.5 ± 53.0	53.4 ± 30.7	0.145
Blood urea nitrogen (mg/dl)	13.9 (10.5–16.9)	17.8 (15.4–27.8)	14.4 (11.6–17.6)	**0.006**
Creatinine (mg/dl)	0.9 (0.8–1.0)	1.4 (1.1–1.8)	1.0 (0.8–1.1)	**0.002**
eGFR (MDRD)	78.1 ± 16.5	49.4 ± 23.4	75.8 ± 23.5	**0.002**
eGFR (MDRD) after 1 year	82.7 ± 16.5	35.1 ± 18.1	72.8 ± 25.6	**< 0.001**
Acute renal failure	3 (6.7)	7 (63.6)	10 (17.9)	**< 0.001**
Glucose (mg/dl)	98.0 (89.0–108.0)	104.0 (99.5–121.5)	100.0 (90.0–112.0)	0.075
Albumin (g/dl)	4.4 (3.9–4.6)	3.8 (3.5–4.2)	4.3 (3.8–4.5)	0.102
CRP (mg/dl)	10.2 (3.7–38.2)	45.3 (19.3–65.5)	15.0 (5.0–44.2)	0.090
Microscopic hematuria	19 (42.2)	9 (81.8)	28 (50.0)	**0.019**
Gross hematuria	14 (31.1)	5 (45.5)	19 (33.9)	0.481
Pyuria	29 (64.4)	8 (72.7)	37 (66.1)	0.732
Proteinuria	6 (13.3)	5 (45.5)	11 (19.6)	**0.029**

Bold values indicate statistically significant differences

Data were presented as mean ± SD, number (percentage) or median (IQR). All data are based on the results at the time of diagnosis except for ‘eGFR after 1 year’

*GUTB* genitourinary tuberculosis, *CKD* chronic kidney disease, *AFB* acid-fast bacilli, *TB* tuberculosis, *PCR* polymerase chain reaction, *ESR* erythrocyte sedimentation rate, *eGFR* estimated glomerular filtration rate, *MDRD* Modification of Diet in Renal Disease study equation, *CRP* c-reactive protein, *SD* standard deviation, *IQR* interquartile range

The sex ratio and BMI did not show significant differences between the two groups. The site of TB infection, previous TB history and comorbidities were not significant independent factors. Treatment modality and duration did not differ significantly between the two groups. Surgical treatment including nephrectomy may be performed during medical treatment, so it does not affect creatinine at the time of diagnosis but may affect CKD development. Of the patients, 15/56 (26.8%) had total or partial nephrectomy; there were no significant differences between the two groups according to kidney function (*p* = 0.674). But, the rate of ESRD development (0.0% vs. 36.4%; *p* = 0.001) and the rate of surgical treatment (20.0% vs. 54.5%; *p* = 0.005) after completion of treatment were significantly higher in the CKD group than in the non-CKD group (Table 3).

**Table 3:** Treatment modality and complications of both groups according to kidney function in GUTB patients

Variables	Normal kidney function group (*n* = 45)	CKD group (*n* = 11)	Total (*n* = 56)	*p*
Medical treatment				
TB medication use	42 (93.3)	11 (100.0)	53 (94.6)	0.894
Medication regimen				1.000
HER	6 (13.3)	1 (9.1)	7 (12.5)	
HERZ	36 (80.0)	10 (90.9)	46 (82.1)	
Medication durations (m)	9 (6–9)	9 (9–10)	9 (6–10)	0.243
Surgical treatment				
All surgical techniques^a^	32 (71.1)	10 (90.9)	42 (75.0)	0.332
Ablative surgery^b^	23 (51.5)	8 (72.7)	31 (55.4)	0.340
Nephrectomy	11 (24.4)	4 (36.4)	15 (26.8)	0.674
Reconstructive therapy^c^	12 (26.7)	3 (27.3)	15 (26.8)	1.000
Follow up durations (m)	38.2 ± 27.8	48.0 ± 47.0	40.5 ± 32.5	0.362
Complications				
Hydronephrosis	9 (20.0)	3 (27.3)	12 (21.4)	**0.038**
Bladder contraction	3 (6.7)	2 (18.2)	5 (8.9)	**0.021**
Renal calcification	10 (22.2)	3 (27.3)	13 (23.2)	**0.044**
Additional surgical treatment^d^	9 (20.0)	6 (54.5)	15 (26.8)	**0.005**
Recurrence	3 (6.7)	1 (9.1)	4 (7.1)	0.056
All cause mortality	2 (4.4)	2 (18.2)	4 (7.1)	0.132
ESRD development	0 (0.0)	4 (36.4)	4 (7.1)	**0.001**

Bold values indicate statistically significant differences

Data were presented as mean ± SD, number (percentage) or median (IQR)

*GUTB* genitourinary tuberculosis, *CKD* chronic kidney disease, *TB* tuberculosis, *HER* isoniazid, rifampicin, and ethambutol, *HERZ* isoniazid, rifampicin, ethambutol, with pyrazinamide, *ESRD* end-stage renal disease, *SD* standard deviation, *IQR* interquartile range

^a^All surgical techniques included all ablative surgery and reconstructive surgery from the time of diagnosis to completion of medical treatment

^b^Ablative surgery is associated with partial or total nephrectomy, nephro-ureterectomy, cystectomy, epididymectomy, semicastration, salpingectomy, as well as other procedures

^c^Reconstructive therapy is considered for: ureteric or urethral stricture repair; stent placement, replacement, or reimplantation; resection; urinary diversion; and bladder augmentation cystoplasty

^d^Any surgical treatment during follow up after completed medical treatment

In the multivariate analysis, ARF (OR, 54.305; 95% CI, 1.517–1944.002; *p* = 0.032) and old age (OR, 54.255; 95% CI, 1.523–1932.905; *p* = 0.028) were independent risk factors for CKD in GUTB patients (Table 4).

**Table 4:** Multivariable analysis of risk factors associated chronic kidney disease in GUTB patients

Variables	Univariate analysis	*p*	Multivariable analysis	*p*
	OR (95% CI)		OR (95% CI)	
Old age (≥65 years)	5.42 (1.25–23.45)	0.029	54.26 (1.52–1932.91)	**0.028**
Acute renal failure	24.50 (4.49–133.76)	< 0.001	54.31 (1.52–1944.00)	**0.032**
Positivity of urine TB PCR study	8.94 (1.01–79.05)	0.030	5.56 (0.19–162.56)	0.320
Microscopic hematuria	6.16 (1.19–31.82)	0.019	13.78 (0.61–313.93)	0.100
Proteinuria	5.42 (1.25–23.45)	0.029	0.30 (0.01–12.85)	0.533

Bold values indicate statistically significant differences

*GUTB* genitourinary tuberculosis, *OR* odds ratio, *CI* confidence interval, *TB* tuberculosis, *PCR* polymerase chain reaction

## Discussion

In this study, we report a high incidence of CKD after treatment in patients whose renal function was normal before the diagnosis of GUTB. Additionally, ARF and old age were independent risk factors for CKD in GUTB patients. This finding suggests that regular follow-up of renal function is needed during and after completion of treatment in elderly GUTB patients with an elevated creatinine level at the time of diagnosis.

CKD is a major global health issue and is ranked 17th in terms of disability-adjusted life years in the US [13]. The overall prevalence of CKD among the general population is 13.1% in the US and 10.8% in China [14, 15]. In Korea, the prevalence of CKD among adults aged >20 years was 8.2%, and that among urban civilians aged >35 years was 13.7% [16, 17]. In our study, the prevalence of CKD in GUTB patients was 19.6%, higher than that in the general population. Likewise, a 19.0% prevalence of renal function deterioration in GUTB patients was reported previously [10]. In our study, ESRD occurred in 7.1% of GUTB patients. According to a review of 8961 cases, GUTB led to ESRD in 5.7% of patients [5]; that prior study involved patients aged 40–45 years, whereas our patients were older (mean, 52.8 ± 16.6 years), which may explain the higher incidence of ESRD [5, 18].

In this study, old age was an important risk factor for CKD in GUTB patients. The eGFR decreases in parallel with age [19]. Renal aging is a multifactorial process associated with anatomical and functional changes that accumulate over a lifetime [20]. Exposure to chronic inflammation, such as that induced by TB, likely enhances oxidative stress and endothelial dysfunction, which are related to renal aging. In addition, the renal-aging-induced reduction in kidney repair ability and tubular and glomerular changes aggravate the decrease in eGFR [20]. Elderly patients with GUTB require close monitoring for early detection of CKD.

A high creatinine level at the time of diagnosis reflects the progression of GUTB and destruction of the kidney structure. After treatment, recovery of a destroyed kidney is difficult. *M. tuberculosis* spreads from the kidney to the bladder, causing granulomatous lesions associated with fibrosis. In the kidney, *M. tuberculosis* forms granulomas in the medullary region and can subsequently disseminate and destroy the renal parenchyma. Papillary necrosis eventually invades the collecting system, leading to fibrosis and obstructive uropathy [21, 22]. Throughout the disease course, papillary necrosis, tubular injury and obstructive uropathy can lead to CKD. In our study, ARF was significantly predictive of CKD and may be related to disease severity at the time of diagnosis. The 10 patients with ARF underwent ablative surgery or reconstruction because of direct infection of the kidney parenchyma and/or ureteral obstruction. Moreover, 10 of the 11 patients in whom CKD developed underwent ablative surgery or reconstruction because of direct infection of the kidney parenchyma and/or ureteral obstruction. Therefore, CKD can be caused by direct infection of the kidney parenchyma or ureteral obstruction with resultant hydronephrosis.

In South Korea, GUTB comprises 3% of EPTB cases. However, GUTB reportedly accounts for 27% (range, 14–41%) of EPTB cases in the US, Canada and United Kingdom [4]. This difference may be due to the low prevalence (<1%) of HIV infection in South Korea. In addition, South Korea has a higher incidence of TB than those of other countries and a high income level, but an intermediate incidence of TB. Thus, further research is needed.

There was no significant difference in the incidence of drug-related side effects, treatment duration or drug regimens between the two groups; thus, such drugs likely did not affect kidney function to an appreciable degree. In univariate analyses, urine TB positivity detected by PCR was associated with CKD. We presumed that the results of this test reflect disease severity and the amount of excretory bacilluria. Delayed diagnosis could aggravate destruction of the GU system, leading to CKD [10, 23]. Therefore, a more sensitive diagnostic method is needed. The diagnosis was confirmed most frequently by histopathology, but urine AFB staining for *M. tuberculosis* was the first-choice diagnostic method in almost all of the patients. Similar to previous reports, the sensitivity of urine AFB testing was very low [21, 23].

Of the patients, 75.0% underwent surgery, similar to previous reports [5, 18]. Furthermore, the CKD group had a significantly higher incidence of additional surgical procedures and ESRD during follow up. Therefore, prevention of CKD development would reduce the need for unnecessary surgical procedures and dialysis. This will require considerable efforts to ensure early diagnosis of GUTB, including the development of a highly sensitive diagnostic tool and active testing of patients.

This study had several limitations. The first was its retrospective design. Second, some patients were lost to follow up, and some had been diagnosed and started treatment at another hospital and were subsequently transferred to our hospital. Third, 14 patients (20%) were excluded because of insufficient information. Fourth, proteinuria was detected by the dipstick test, which is not quantitative. Fifth, the single-centre design and small sample size may limit the generalisability of our findings to the overall population. However, previous literature reviews comprise mainly case reports or hypothesised that CKD is related to GUTB. Thus, this study is the first to analyse the risk factors for CKD in GUTB patients.

## Conclusions

ARF and old age are independent risk factors for CKD in GUTB patients. Therefore, in elderly GUTB patients with an elevated creatinine level at the time of diagnosis, regular follow-up of renal function should be performed during and after treatment.

## Abbreviations

AFB: Acid-fast bacilli
ARF: Acute renal failure
BMI: Body mass index
CI: Confidence interval
CKD: Chronic kidney disease
eGFR: estimated glomerular filtration rate
EPTB: Extrapulmonary tuberculosis
ESRD: End-stage renal disease
GU: Genitourinary
GUTB: Genitourinary tuberculosis
HIV: Human immunodeficiency virus
IQR: Interquartile range
KDIGO: Kidney Disease: Improving Global Outcomes
*M. tuberculosis*: *Mycobacterium tuberculosis*
MDRD: Modification of Diet in Renal Disease study equation
OR: Odds ratio
PCR: Polymerase chain reaction
TB: Tuberculosis
